# Fasudil, a Clinically Used ROCK Inhibitor, Stabilizes Rod Photoreceptor Synapses after Retinal Detachment

**DOI:** 10.1167/tvst.6.3.22

**Published:** 2017-06-20

**Authors:** Ellen Townes-Anderson, Jianfeng Wang, Éva Halász, Ilene Sugino, Amy Pitler, Ian Whitehead, Marco Zarbin

**Affiliations:** 1 Department of Pharmacology, Physiology, and Neuroscience, Rutgers New Jersey Medical School, Newark, NJ, USA; 2 Institute of Ophthalmology and Visual Science, Rutgers New Jersey Medical School, Newark, NJ, USA; 3 Department of Microbiology, Biochemistry, and Medical Genetics, Rutgers New Jersey Medical School, Newark, NJ, USA

**Keywords:** synaptic plasticity, ROCK inhibition, photoreceptor synapse, retinal detachment, RhoA signaling

## Abstract

**Purpose:**

Retinal detachment disrupts the rod-bipolar synapse in the outer plexiform layer by retraction of rod axons. We showed that breakage is due to RhoA activation whereas inhibition of Rho kinase (ROCK), using Y27632, reduces synaptic damage. We test whether the ROCK inhibitor fasudil, used for other clinical applications, can prevent synaptic injury after detachment.

**Methods:**

Detachments were made in pigs by subretinal injection of balanced salt solution (BSS) or fasudil (1, 10 mM). In some animals, fasudil was injected intravitreally after BSS-induced detachment. After 2 to 4 hours, retinae were fixed for immunocytochemistry and confocal microscopy. Axon retraction was quantified by imaging synaptic vesicle label in the outer nuclear layer. Apoptosis was analyzed using propidium iodide staining. For biochemical analysis by Western blotting, retinal explants, detached from retinal pigmented epithelium, were cultured for 2 hours.

**Results:**

Subretinal injection of fasudil (10 mM) reduced retraction of rod spherules by 51.3% compared to control detachments (*n* = 3 pigs, *P* = 0.002). Intravitreal injection of 10 mM fasudil, a more clinically feasible route of administration, also reduced retraction (28.7%, *n* = 5, *P* < 0.05). Controls had no photoreceptor degeneration at 2 hours, but by 4 hours apoptosis was evident. Fasudil 10 mM reduced pyknotic nuclei by 55.7% (*n* = 4, *P* < 0.001). Phosphorylation of cofilin and myosin light chain, downstream effectors of ROCK, was decreased with 30 μM fasudil (*n* = 8–10 explants, *P* < 0.05).

**Conclusions:**

Inhibition of ROCK signaling with fasudil reduced photoreceptor degeneration and preserved the rod-bipolar synapse after retinal detachment.

**Translational Relevance:**

These results support the possibility, previously tested with Y27632, that ROCK inhibition may attenuate synaptic damage in iatrogenic detachments.

## Introduction

Detachment is well-known to affect synapses in the outer plexiform layer (OPL).^1,2^ Synaptic injury begins with retraction of the rod presynaptic terminals towards their cell bodies. Axonal retraction results in the disjunction of the first synapse in the visual pathway as the rod presynaptic terminal disconnects from postsynaptic bipolar dendrites.^3^ Cone terminals also are disrupted; they lose their synaptic invaginations and normal connections with bipolar cells.^2,4^ The retraction of rod terminals is followed after a short period by extension of bipolar dendrites into the outer nuclear layer (ONL) and sprouting from horizontal cells.^2^ Horizontal cell processes sometimes extend well into the subretinal space. Although this pathology originally was described in animal models, the same features, rod axon retraction and bipolar and horizontal cell sprouting, have been reported in humans after detachment.^4,5^

Reattachment allows the photoreceptor outer segments (OS) to regrow.^6^ However, it does not restore retinal synaptic structure completely.^7^ In fact, additional anomalies occur, including inappropriate photoreceptor sprouting from rod terminals into the inner nuclear layer (INL). Approximately a quarter to one-half of patients with either macula-on or macula-off detachments, respectively, do not recover visual function to levels equivalent to the fellow eye, even with anatomically successful surgery.^8–11^ It has been suggested that the lack of visual recovery after reattachment can be attributed in part to continued disruption of synaptic connectivity.^4,12^ Thus, preservation of synaptic structure in the injured retina may have significant therapeutic benefit.

**Figure 1:**
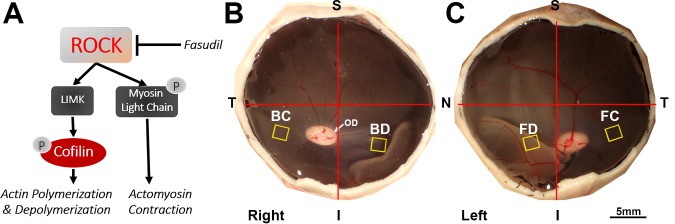
(A) Proposed mechanism for fasudil inhibition of photoreceptor axonal retraction. Fasudil inhibits ROCK, which reduces LIMK activity and phosphorylation of cofilin and MLC. Phosphorylation of cofilin and MLC cause actin turnover and actomyosin contraction, which leads to axon retraction. (B, C) Location of iatrogenic retinal detachments and sample collection. Retinal detachments were created in the inferior nasal quadrant after vitrectomy. Samples from the *yellow square areas* were used for morphological anyasis. (B) Control eye. BD, detached area, detached with subretinal injection of BSS; BC, attached area. (C) Treated eye. FD, detached area, detached with subretinal injection of fasudil in BSS; FC, attached area; T, temporal; N, nasal; S, superior; I, inferior; OD, optic disc.

We have focused on the synaptic changes in rod terminals, since they are morphologically striking and relatively easy to monitor. Examining first isolated salamander photoreceptors in culture, then retinal explants from adult pigs, and finally iatrogenically-produced detachments in living pigs, we demonstrated that activation of the RhoA signaling pathway leads to axonal retraction by rod cells (Fig. 1A).^13–15^ We hypothesized that ROCK inhibition could reduce axonal retraction. Initially, we used Y27632, an experimental ROCK inhibitor that binds competitively to the ATP binding site of ROCK and, thus, prevents kinase activity. It inhibits both isoforms of ROCK, I and II. When applied to isolated photoreceptors, to retinal explants, or to the subretinal space of detached retinae in vivo, Y27632 consistently reduced the amount of rod axon retraction.^13–15^ Based on these findings, we sought to examine the effectiveness of a clinically approved ROCK inhibitor, fasudil, that also inhibits ROCK I and II by binding to the ATP binding site.^16^

Our initial experiments in the living pig demonstrated that synaptic change occurs very rapidly after injury. Within the first 2 hours after detachment, axonal retraction has begun. There was no evidence of bipolar dendritic sprouting at this time point, demonstrating that rod-bipolar connections were breaking quickly.^15^ Therefore, in this report, we have chosen to assess early changes, that is, at 2 hours after the detachment injury.

## Materials and Methods

### Animals

Pig eyes are similar in size and vascular anatomy to the human eye, and porcine retina contains rod and cone cells.^17–19^ Although there is no fovea in the pig, there is an area centralis that is rich in cones. Thus, experiments on adult pigs are useful for translational work.

Female Yorkshire pigs, 3 months old and weighing 30 kg, were obtained from Animal Biotech Industries (Danboro, PA). Pigs were kept on a 12-hour light/12-hour dark cycle for at least 2 days before surgery in an Association for Accreditation and Assessment of Laboratory Animal Care (AAALAC)–accredited pathogen-free facility. The animals were subject to overnight fasting and ad lib access to water before surgery. A total of 16 animals (32 eyes) were used.

Pig eyes used to create retinal explants were obtained from a local abattoir through Animal Parts (Scotch Plains, NJ). The male/female Yorkshire pigs, 6 months old and weighing 60 to 75 kg, were sacrificed mid-day; the eyes were kept on ice and delivered to the lab within 2 hours. A total of 9 eyes were used.

Experimental procedures and methods of euthanasia were approved by the New Jersey Medical School Institutional Animal Care and Use Committee and adhered to the ARVO Statement for the Use of Animals in Ophthalmic and Vision Research.

### Retinal Detachment Procedures and Drug Administration

For retinal detachments in vivo, animals were premedicated with atropine (0.02 mg/kg; VetUS, Henry Schein, Dublin, OH) and anesthetized with ketamine (20 mg/kg; Mylan Institutional LLC, Galway, Ireland) and xylazine (2.2 mg/kg; LLOYD Lab., Shenandoah, IA) injected intramuscularly, and then catheterized and intubated. Animals were administered pre- and postoperative intravenous injections of buprenorphine (0.01–0.05 mg/kg; Reckitt Benckiser HealthCare, Hull, England) and enrofloxacin (10 mg/kg; Bayer HealthCare, Shawnee, KS). To maintain anesthesia, the animals were given 1% to 3.0% isoflurane in oxygen using a ventilator. Lactated Ringer's solution was infused intravenously at a rate of 8 mL/kg/h. Under anesthesia, pupils were dilated with topical application of 1% tropicamide (Bausch & Lomb, Tampa, FL) and 2.5% phenylephrine hydrochloride (Paragon Bioteck, Portland, OR). A standard 3-port vitrectomy was done using 20-gauge instrumentation. The posterior hyaloid was elevated off the area centralis using active suction, and a core vitrectomy was completed. During and after vitrectomy, the vitreous cavity of the eye was perfused with a mammalian balanced salt solution (BSS; Alcon, Fort Worth, TX) containing 1 μg/mL epinephrine (Henry Schein, Dublin, OH). A bent 33-gauge metal cannula with a 50 to 100 μm tip was used to slowly inject BSS or fasudil (Selleckchem, Boston, MA; dissolved in BSS) subretinally to create a retinal detachment (∼10–15 mm in diameter) in the inferior nasal quadrant (Figs. 1B, 1C). For intravitreal administration, 150 μL of 200 mM fasudil dissolved in BSS was injected with a 30-gauge needle into the vitreous cavity (entering ∼3 mm posterior to the limbus) either immediately after or 2 hours after retinal detachment surgery. After the eye was treated with drug for 2 hours, the animal was sacrificed for enucleation. Eyes were kept in ice-cold Dulbecco's Modified Eagle's Medium (DMEM) containing 4.5 g/L glucose, L-glutamine and sodium pyruvate (10–013 CV; Cellgro, Mediatech, Manassas, VA) until opened to collect samples, usually approximately 20 minutes later. Once the eyes were opened, the anterior segment and any remaining vitreous humor were removed carefully, and a few drops of DMEM were added to keep the surface of the neural retina moist. The neural retinal tissues were collected as diagrammed in Figure 1 after fixation in 4% paraformaldehyde (EMS, Hatfield, PA) in 0.125 M phosphate buffer (PB; pH 7.4) for morphological analysis.

### Western Blotting

Explants of detached retina were created as described previously.^15^ Briefly, after the surrounding orbital tissue was removed, the eyes were washed twice with DMEM. The anterior segment and vitreous body were removed carefully, and a few drops of DMEM were added to keep the surface of the neural retina moist. Buttons of retinal tissue (6 mm) were created using a trephine and detached from the underlying retinal pigment epithelium by injecting a few drops of DMEM along the cut edge. The detached neural retinae were removed gently and cultured in Neurobasal-A media (10888-022; Gibco, Life Technologies, Grand Island, NY) supplemented with 1% GlutaMAX-I (Gibco), 2% B-27 Supplement (Gibco), and 100U/mL of penicillin and 100 μg/mL streptomycin (Gibco) at 37°C for 2 hours, and then snap-frozen with dry ice plus ethanol and stored at −80°C for further use.

Trephined retinae (6-mm diameter buttons) were lysed, homogenized in 1 × RIPA buffer (EMD Millipore, Billerica, MA) containing 1× protease inhibitor (Roche, New York, NY), 2 mM Na_3_VO_4_, and 10 mM NaF with Ceria Stabilized Zirconium Oxide Beads (0.5 mm in diameter, ZrOB05; Next Advance, Averill Park, NY), and centrifuged at 15,000*g* for 10 minutes at 4°C. Protein concentrations were determined using Quick Start Bradford Protein Assay Kit (Bio-Rad, Hercules, CA). The samples then were dispersed in an equal volume of 2 × SDS loading buffer (Bio-Rad) containing 10% 2-mercaptoethanol, boiled for 5 minutes, electrophoresed with 10% or 8% to 16% precast polyacrylamide gels (Bio-Rad), transferred to a nitrocellulose membrane (0.2 μm, Bio-Rad), and analyzed by Western blotting. The following antibodies were used: phospho-Myosin Light Chain 2 (pMLC, Ser 19) mouse monoclonal antibody (mAb), and cofilin, phospho-cofilin (p-cofilin), and Myosin Light Chain 2 (MLC) rabbit mAbs (all from Cell Signaling, Boston, MA). For an internal control, glyceraldehyde-3-phosphate dehydrogenase rabbit pAb (GAPDH; Santa Cruz Biotechnology, Dallas, TX) was blotted in the same membrane. The membranes were visualized using secondary goat anti-mouse or rabbit IgG antibody conjugated to horseradish peroxidase (Jackson ImmunoResearch Laboratories, West Grove, PA). Forte Western HRP substrates (EMD Millipore) were used to visualize the immune complexes. The density of a specific band was determined using ImageJ (v1.45s, National Institutes of Health [NIH], Bethesda, MD).

### Immunohistochemistry

Retinal samples were fixed overnight at 4°C and then immersed in 30% sucrose for an additional night at 4°C, embedded and frozen in OCT compound (Sakura Finetek, Torrance, CA), and cut into 15-μm thick sections using a cryostat as described previously.^15^ Sections were immunolabeled for SV2 (Developmental Studies Hybridoma Bank, Iowa City, IA) with secondary antibodies conjugated to Alexa Fluor 488 (Life Technologies, Norwalk, CT), followed by propidium iodide (PI; 1 μg/mL; Sigma-Aldrich Corp., St. Louis, MO) to stain nuclei. Labeled sections were covered with Fluoromount-G medium (SouthernBiotech, Birmingham, AL) and preserved under coverslips sealed with nail polish. Sections were examined with a confocal microscope (LSM510; Carl Zeiss Microscopy, Jena, Germany) by scanning 1.0 μm optical sections with a ×63 oil immersion objective. Control sections were processed simultaneously with experimental sections but without primary antibodies.

### Quantification of Axonal Retraction

All data were collected by researchers masked to the sample identifications. Brightness and contrast were set to obtain unsaturated images. Laser power and scan rate were unchanged throughout a single experiment. Enhancements in brightness and contrast were performed (Photoshop 7.0; Adobe, Mountain View, CA) only for presentation purposes. SV2 immunolabeling was analyzed as described previously.^15^ Briefly, a binary mask was created for each image and the ONL was outlined; all labeled pixels in the ONL were counted using ImageJ (v1.45s; NIH). The measurements are reported as pixels per micrometer of the ONL length. Data were collected from two to four sections per specimen examining at least three different areas of each section.

### Statistical Analysis

All data were tested with a paired Student's *t-*test. Use of the paired *t*-test was based on the experimental design. For images, data from the same region of right and left eyes of the same animal or from detached and attached regions of the same retina were paired. The use of the paired *t*-test assumed linearity of the data and a normal distribution of the paired differences. For Western blots, explants from the same eye were compared. The fixed normalization point technique was used; control data were set at 100%.^20^ For data derived from examination of tissue sections, no normalization was done. Statistical analysis was performed with Sigma Plot (version 11, Chicago, IL) or GraphPad Prism (v5.0, La Jolla, CA). The graphics were produced using GraphPad Prism. Data are expressed as mean ± SD. We set α (type I error rate) at 0.05.

## Results

### Subretinally Administered Fasudil Reduced Rod Photoreceptor Axonal Retraction

**Figure 2:**
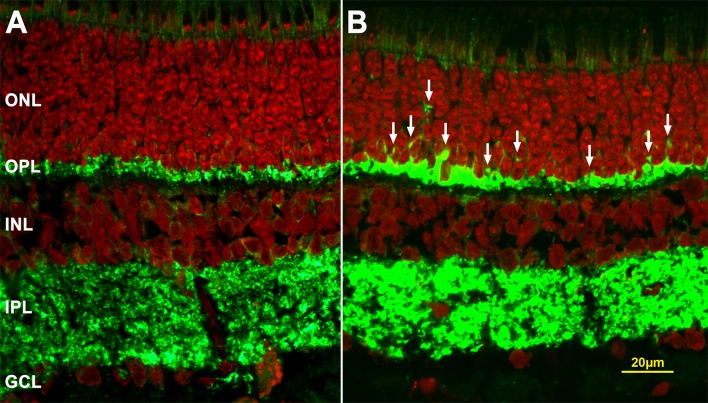
Axonal retraction by rod photoreceptors after 2 hours of detachment. (A) The normal retina, labeled for synaptic membrane protein (SV2, *green*) and nuclei (stained with PI, *red*). SV2 label is present in the OPL and IPL. (B) Two hours after detachment SV2-immunolabel appears in the ONL. This eye received a subretinal injection of 1 mM fasudil during creation of the detachment. SV2-labeled spots (*white arrows*) in the ONL indicate axonal retraction. Other retinal layers appear normal. INL, inner nuclear layer; GCL, ganglion cell layer.

Normally, SV2-immunolabeling is observed only in the inner segments (IS) of photoreceptors and the synapses in the OPL and inner plexiform layers (IPL), but after retinal detachment, SV2-labeling spreads into the ONL (Figs. 2A, 2B). Label in the ONL appears in rod cell somata or as discrete puncta similar in size to an individual rod spherule. Label in the ONL is due to retraction of the rod axon terminal and axon.^2,15^ Although cone synapses are affected by detachment, their axon terminals do not retract.

In the past, ROCK inhibition with Y27632 reduced the amount of retraction observed in the ONL by rod photoreceptors in vitro and in vivo.^13–15^ The question we addressed in the present experiments is whether fasudil also can reduce synaptic disruption. For these experiments, we used a live pig retinal detachment model as described in our previous study.^15^ BSS was used to create a retinal detachment in one eye as a control, and 1 or 10 mM fasudil dissolved in BSS was used to create a retinal detachment in the fellow eye in the corresponding nasal inferior retinal quadrant (Figs. 1B, 1C). Doses were based on previous success with 1 and 10 mM Y27632. The eyes were removed and fixed after 2 hours. SV2-labeling in the different retinal areas (BC and BD, attached and detached areas, respectively, in the eye using BSS for detachment; FC and FD, attached and detached areas, respectively, in the eye using fasudil for detachment) were compared (Fig. 1).

With 1 mM fasudil treatment, we detected no difference in axonal retraction in the treated detached area (FD) compared to the untreated detached area (35.1, average number of labeled pixels per μm of ONL length in FD and BD; *n* = 4 pigs, 400 images; see Fig. 2B for detached retina treated by 1 mM fasudil). Additionally, there was no difference between detached and attached retinal areas within each eye, treated and control. Thus, 1 mM fasudil had no effect on retraction. However, as reported previously,^15^ retraction occured quickly, that is by 2 hours after detachment, and was present where the detachment occurred as well as in regions several millimeters away from the injury.

**Figure 3:**
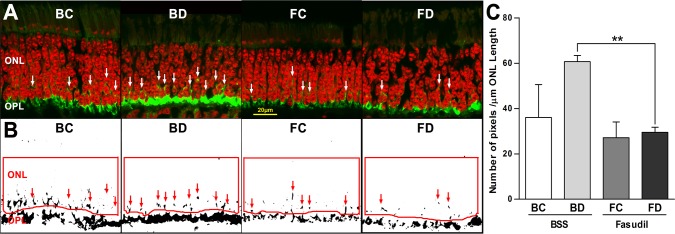
Effect of subretinal injection of 10 mM fasudil on axonal retraction by photoreceptors 2 hours after retinal detachment. (A) Representative images of retina labeled for SV2 (*green*) and nuclei (*red*). Control eye: attached (BC) and detached (BD) retinae. Treated eye: attached (FC) and detached (FD) retinae treated with 10 mM fasudil. SV2-labeled spots (*white arrows*) in the ONL indicate axonal retraction. (B) Binary images created from the *green* channel from images in (A). SV2-labeled spots are indicated with *red arrows*. (C) Comparison of SV2-labeled pixels/μm ONL length in different retinal areas. There was a significant reduction of SV2-labeled pixels, by 51.3%, in FD (29.2 ± 2.7 pixels/μm ONL) compared to BD (60.7 ± 2.7 pixels/μm ONL) indicating a reduction of axon retraction (**P = 0.002, *n* = 3 animals, 300 images, ± SD). There was no significant difference between BC and BD in SV2-labeled pixels.

When the concentration of fasudil was increased to 10 mM, axon retraction was reduced. The number of SV2-labeled pixels per μm ONL length was decreased significantly in the treated detached retina (FD) by 51.3% compared to the untreated detached retina (BD; Fig. 3). Axonal retraction was decreased by 24.7% in the attached retina of the treated eye (FC) compared to the corresponding area, BC, in the control eye, but the reduction was not statistically significant. Within a single eye, the amount of SV2 labeling in the attached versus detached areas (BD versus BC and FD versus FC) also was not different; although area BD had SV2-labeling in the ONL that was 40.5% higher than area BC, this was not statistically significant. Thus, a subretinal injection of 10 mM fasudil primarily reduced axon retraction in the detached retina where the reduction was profound.

### Fasudil Administered Intravitreally Reduced Rod Photoreceptor Axonal Retraction

**Figure 4:**
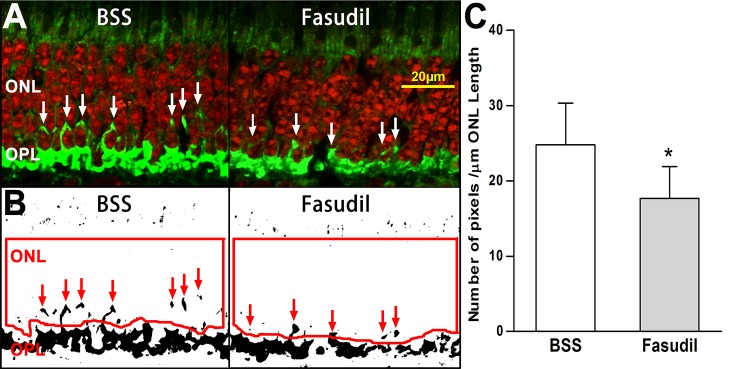
Effect of intravitreal injection of fasudil on axonal retraction by photoreceptors 2 hours after retinal detachment. (A) Representative images of retina labeled for SV2 (*green*) and nuclei (*red*). Control eye, detachment made with BSS; treated eye, detachment made with BSS, fasudil injected immediately afterward intravitreally to achieve a 10 mM concentration. SV2-labeled spots (*white arrows*) in the ONL indicate axonal retraction. (B) Binary images created from the *green* channel from (A). SV2-labeled spots indicated with *red arrows*. (C) Comparison of the average of attached and detached areas between BSS control and fasudil-treated eye. The axonal retraction was significantly reduced by 28.7% in fasudil-treated eyes compared to BSS (*P < 0.05, *n* = 5 animals, 500 images, ± SD).

Clinically, intravitreal injection of drugs is more straightforward than subretinal injection, which requires specialized equipment and may cause other complications. Therefore, we also applied fasudil with an intravitreal injection to test for reduction of photoreceptor axonal retraction after retinal detachment. The volume of the eye was determined by filling the posterior eyecup with liquid after removal of the anterior segment and vitreous as shown in Figure 1B. The volume of the eye cup was approximately 3 mL for a 30 kg Yorkshire pig. To achieve an effective dose of 10 mM, 150 μL of 200 mM fasudil was injected intravitreally using a 30-gauge needle approximately 3 mm posterior to the limbus immediately after retinal detachment. Two hours after treatment, the pig was enucleated and the retinae were prepared for histology. Because the intravitreal injections presumably allowed drug to reach detached and attached retinae equally, we combined the data of these two regions (BC, 24.3 ± 9.6; BD, 25.2 ± 9.9; FC, 18.7 ± 8.6; FD, 16.5 ± 6.4; combined BSS, 24.7 ± 5.6; combined Fasudil, 17.6 ± 4.2; all in pixels/μm of ONL length). The number of SV2-labeled pixels/μm of ONL length in the detached and attached area in the treated eye (Fasudil) was significantly less by 28.7% (*P* = 0.04, *n* = 5 animals) than the average value in the control (BSS) eye (Fig. 4). The reduction was smaller than that for the subretinal injection; however, it remained statistically significant when tested with the nonparametric Wilcoxon signed-rank test (*P* = 0.03). The results indicated that immediate treatment with fasudil through intravitreal injection reduces axon retraction after retinal detachment.

### Fasudil Reduced Phosphorylation of Cofilin and MLC in Retinal Explants

**Figure 5:**
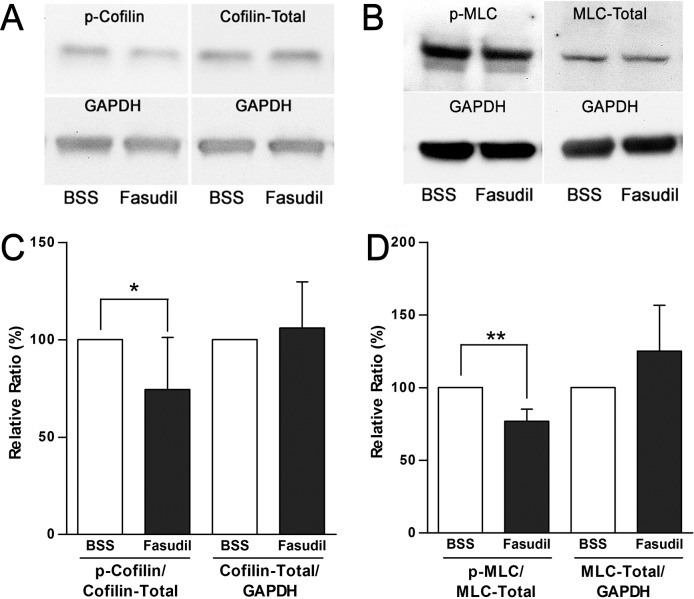
Levels of phosphorylated cofilin (p-cofilin) and MLC (pMLC) after 2 hours of treatment with fasudil (30 μM) in detached retinal explants. (A, B) Typical Western blots using antibodies against p-cofilin, cofilin, pMLC, MLC, and GAPDH. (C) The ratio of p-cofilin/cofilin-Total was reduced significantly with fasudil treatment by 25.8 ± 26.8% (*P < 0.05, *n* = 5 eyes, 10 explants, ± SD), while total cofilin (normalized by GAPDH) did not change. (D) The ratio of pMLC/MLC-Total was significantly reduced by 23.2 ± 8.2% with fasudil (**P < 0.05, *n* = 4 eyes, 8 explants, ± SD), while total MLC (normalized by GAPDH) did not change significantly.

We have shown that the RhoA signaling pathway is activated after retinal detachment in vitro and in vivo;^13–15,21^ ROCK phosphorylates MLC directly and stimulates its downstream effector LIM kinase (LIMK), which in turn phosphorylates cofilin (Fig. 1A). To test whether fasudil was acting by inhibiting ROCK activity, we assayed the phosphorylation of cofilin and MLC in vitro, in pig retinal explants detached from the retinal pigmented epithelium, after a 2-hour treatment with 30 μM fasudil. Western blot analysis showed that the ratio of p-coflin/cofilin-total is significantly reduced by 25.8% (*P* < 0.05, *n* = 5 eyes, 10 explants) in fasudil-treated samples compared to controls (Figs. 5A, 5C), while the amount of total cofilin did not change. For MLC, the ratio of pMLC/MLC was significantly reduced by 23.2% in the fasudil-treated group compared to control, while total MLC was elevated but not significantly (Figs. 5B, 5D). These results indicate that fasudil inhibited the ROCK signaling pathway.

### Fasudil Reduced Rod Cell Death after Retinal Detachment in Delayed Treatment

**Figure 6:**
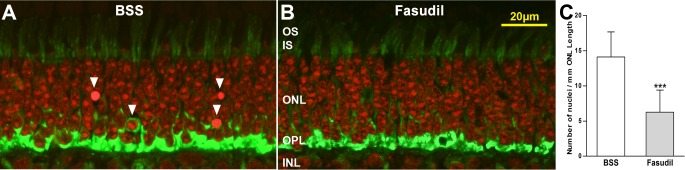
Rod cell degeneration in the detached retina after a 4-hour detachment. Fasudil (10 mM) was injected intravitreally 2 hours after the detachment was created; samples were collected an additional 2 hours later. Nuclei were stained with PI (*red*); synaptic terminals were labeled for SV2 (*green*). (A, B) Representative sections of retina from untreated (BSS) and treated (Fasudil) eyes. Densely stained (pyknotic) nuclei are present in the ONL of the untreated detached retina (*arrowheads*) where rod cell bodies reside. (C) Treatment with fasudil significantly reduced the number of densely stained nuclei in the ONL by approximately 55.7% compared to controls (BSS, 14.1 ± 3.5; fasudil, 6.3 ± 3.1 nuclei/mm ONL; ***P < 0.001, *n* = 4 animals, 400 images, ± SD).

To test whether delayed treatment with fasudil can prevent axonal retraction, 150 μL of 200 mM fasudil (10 mM final concentration) was injected intravitreally 2 hours after retinal detachments were made by subretinal injection of BSS. The animals remained under anesthesia for another 2 hours before they were sacrificed for morphologic analysis. Analysis of axonal retraction showed that there was no significant difference between the fasudil-treated group (24.9 pixels/μm ONL length) and the control group (20.4 pixels/μm ONL length; *n* = 4). However, we discovered that in the retinae detached for 4 hours, unlike retinae detached for only 2 hours,^15^ there were nuclei stained densely by PI, and, therefore, defined as pyknotic, in the ONL in regions occupied by rods (Figs. 6A, 6B). Nuclear condensation is a hallmark of apoptosis, and PI staining has been used to identify apoptosis in retinal detachment.^22^ With 10 mM fasudil treatment the number of pyknotic nuclei was reduced substantially by approximately 55.7% at 4 hours (*P* < 0.001, Fig. 6C). This observation suggested that fasudil was able to reduce rod cell degeneration.

## Discussion

In the normal retina there virtually is no synaptic membrane protein immunolabel in the ONL. However, only 2 hours after detachment, as reported previously,^15^ synaptic protein label, and, thus, axon retraction, appeared in the ONL. In addition, retraction occurred in detached retinae and in tissue several millimeters away. The presence of axon retraction correlates with increases in activated RhoA, which was reported in the detached retina but also in retina outside the detachment.^15^ Thus, these findings confirm our previous analysis, that the injury response to retinal detachment is not confined to the area of detachment but affects large areas of adjacent attached retina within hours.

Fasudil proved to be effective at reducing axon retraction and rod cell death in the short-term experimental rhegmatogenous detachments used here in the adult pig. Subretinal fasudil application compared favorably with the experimental ROCK inhibitor Y27632, which our group had tested previously. However, there were some differences. Although both drugs were used successfully at 10 mM (43.7% reduction in synaptic retraction for Y27632 versus 51.3% for fasudil), Y27632 also reduced axon retraction at 1 mM concentrations by 34.5%.^15^ In addition, Y27632 at the 10 mM concentration reduced axon retraction in detached retina and the distant attached retina. These data suggested that Y27632 may be more efficacious. The K_i_'s of the two drugs used here are similar (140 and 330 nM for Y27632 and fasudil, respectively),^23^ but their half-lives may be different. Nothing is yet known about the half-lives for ROCK inhibitors placed intraocularly, but 10 mg/kg Y27632, when injected intraperitoneally, can cross the blood–brain barrier and has a half-life of 60 to 90 minutes in mouse brain^24^ whereas intravenous injection of fasudil is reported to have a half-life of approximately 40 to 45 minutes in rat and human, respectively, in plasma.^25–27^ A longer half-life for Y27632 would allow time for diffusion of active inhibitor away from the detachment and thereby account for its broader effects. It is known that the half-lives of drugs can be extended by encapsulation in liposomes, and this strategy has been used successfully for intravitreal administration of fasudil.^28^ Thus, in the future, liposomal preparations of fasudil will be tested.

The relatively high concentration of fasudil needed, 10 mM, to significantly reduce axon retraction suggests that in addition to a short systemic half-life, fasudil may be cleared rapidly from the intraocular compartment. However, it also raises the concern that fasudil may be acting on secondary targets, such as protein kinase A (PKA), PKG, PKC, and MLC kinase (MLCK; K_i_s of 1.6, 1.6, 3.3, and 36 μM, respectively)^29^ in addition to ROCK I and II. We confirmed that fasudil was working on the RhoA pathway using retinal explants and testing for levels of phosphorylated cofilin and MLC. The fact that phosphorylation is reduced for both proteins while total protein levels were not significantly altered confirms that fasudil is modulating Rho kinase in the RhoA pathway. However, it does not rule out additional secondary effects. In addition to inhibition of secondary kinases, a recent report describes interactions of fasudil with K_v_7.4 and 7.4/7.5 channels resulting in increased M-type K^+^ currents in vascular tissue.^30^ Although most neurons do not contain these particular channels, rod and cone cells have been shown to contain message and protein for K_v_7.4 and 7.5.^31,32^ The function of these proteins, possibly occurring as channels, in photoreceptors currently is unknown.

Our report included, for the first time to our knowledge, an examination of axon retraction after an intravitreal injection of a ROCK inhibitor. The amount of retraction after the intravitreal injection was less, by 28.7%, than after a subretinal injection, 51.3%; however, the reduction was significant. Therefore, fasudil probably can move through the retina to inhibit rod axon terminals. Although fasudil originally was used for cerebral vasospasm,^33^ intravitreal fasudil already has been applied to patients with diabetic macular edema and optic nerve damage to successfully interrupt vascular pathology.^34,35^ Intravitreal injections of fasudil (10 μM) in patients showed no evidence of intraocular toxicity. Our results, using higher doses, also showed no toxic responses over 2 hours, and, indeed, intravitreal injections after 2 hours of detachment in the pig dramatically decreased the number of pyknotic nuclei in the outer nuclear area in the region of rod cell somata and, thus, had a protective effect on rod photoreceptors for the short term. This result is perhaps not surprising as activated RhoA has been shown to be an inducer of apoptosis,^36^ and application of ROCK inhibitors has been reported to reduce apoptosis in the retina.^37–42^ In the latter study on the RCS rat, a single 10 to 50 mM dose of Y27632 injected intravitreally (1–5 mM estimated final concentration) had the optimal antiapoptotic effect on photoreceptors, a protocol similar to our report. Thus, intravitreal fasudil seems well tolerated by the eye although long-term studies in our model system remain to be done. Because intravitreal injection has a relatively low incidence of complications and is easy to administer in an outpatient environment, it may, in the future, be the preferred route for drug delivery in the setting of retinal detachment.

An unexpected result occurred with fasudil, however, for intravitreal injections after 2 hours of a 4-hour detachment. We had hypothesized that a delayed treatment would decrease retraction of those axons that initiated retraction later. We had demonstrated that Y27632 can reduce rod axon retraction when applied 6 hours after a detachment in an in vitro model of porcine retinal detachment.^13^ However, there was no effect on retraction with a delayed fasudil treatment in vivo, even though the drug was effective at reducing rod cell death. At present the explanation is not clear. The timeline of axon retraction appears slower in vitro^13^ than in vivo.^15^ Possibly, in vivo the initial fast rate of retraction has slowed after 2 hours so an effect is difficult to detect with a short-lived drug. In the cat model of rhematogenous detachment in vivo, synaptic protein label in the ONL is quite abundant in detachments from 1 to 28 days old,^2^ suggesting that retraction continues for several days at least, but the rate of axon retraction is unknown. We have preliminary data that RhoA activation is higher in detached retina than in uninjured retina at 10 and 24 hours, but reduced from its peak at 2 hours (Wang J, Zarbin M, Sugino I, Whitehead I, and Townes-Anderson E. Personal observations, 2016). Changes in RhoA activation over time also have been reported in other injured neural tissue.^43^ Alternatively, downstream kinases, such as LIMK, may be active at 2 hours, and their activity would not be inhibited by fasudil (Fig. 1A). We have shown previously that LIMK contributes to axon retraction in pig retina^21^ and that using inhibitors to ROCK and LIMK has an increased inhibitory effect on axon retraction. It will be instructive to apply an inhibitor to LIMK in a delayed treatment scenario after detachment. Future experiments should be able to resolve these questions.

Our results suggested that there are several possible retinal applications of fasudil. Fasudil could be useful for iatrogenic retinal detachments, such as detachments created for subretinal delivery of stem cells, viral vectors, or visual prostheses. Since the timing of the detachment is predetermined in these cases, it should be possible to inject fasudil into the subretinal or intravitreal space either before or during creation of the detachment. The drug would protect the OPL from damage. The potential value of stabilizing the outer retinal circuitry during iatrogenic detachments is supported by a recent report that described changes in the outer synaptic layers of an enucleated human eye after subretinal placement of a visual prosthesis (Chen J, et al. *IOVS* 2016;57:ARVO E-Abstract 3732). The disruption reported was likely due to axon retraction. In addition, a recent report described retinal recovery after iatrogenic macular detachment for gene therapy in five patients with choriodemia.^44^ Although OCT showed full reattachment, visual recovery was not uniform among the patients. Sensitivity and color vision did not return to baseline after 1 month for approximately a third of the treated eyes. Again, it is possible that OPL damage had occurred. Although we have yet to demonstrate directly that morphologic disruption of the OPL leads to functional problems, anatomy and physiology are closely linked. Therefore, we propose that the success of subretinal procedures may be enhanced by treatment with ROCK inhibitors. Intravitreal fasudil may be additionally useful in retinal degenerations to reduce cell death and in degenerations that exhibit rod axon retraction pathology (RCS rat^45^; nob2 mouse, a model of congenital stationary night blindness^46^; retinoschisis knockout mouse^47^; rat model of oxygen-induced retinopathy^48^). The retraction observed in these degenerations is morphologically the same as that seen in iatrogenic retinal detachment. If, like detachment, it is caused by RhoA activation, ROCK inhibition may help preserve outer synaptic connections.

There are several avenues that could be pursued to better understand the breadth of possible applications of ROCK inhibitors to retinal injury and disease. Fortuitously, a number of ROCK inhibitors are being tested for ocular use in clinical trials.^49^ Their binding constants, half-lives, off-target effects, solubility, and so forth vary. If these drugs prove useful in patient treatment, they can be repurposed and tested as agents for retinal protection.
